# 22q11.2 Low Copy Repeats Expanded in the Human Lineage

**DOI:** 10.3389/fgene.2021.706641

**Published:** 2021-07-15

**Authors:** Lisanne Vervoort, Nicolas Dierckxsens, Zjef Pereboom, Oronzo Capozzi, Mariano Rocchi, Tamim H. Shaikh, Joris R. Vermeesch

**Affiliations:** ^1^Department of Human Genetics, KU Leuven, Leuven, Belgium; ^2^Centre for Research and Conservation, Royal Zoological Society of Antwerp, Antwerp, Belgium; ^3^Evolutionary Ecology Group, Department of Biology, Antwerp University, Antwerp, Belgium; ^4^Department of Biology, University of Bari, Bari, Italy; ^5^Section of Genetics and Metabolism, Department of Pediatrics, University of Colorado School of Medicine, Aurora, CO, United States

**Keywords:** 22q11.2 deletion syndrome, segmental duplication, structural variation, chromosome 22 evolution, low copy repeats

## Abstract

Segmental duplications or low copy repeats (LCRs) constitute duplicated regions interspersed in the human genome, currently neglected in standard analyses due to their extreme complexity. Recent functional studies have indicated the potential of genes within LCRs in synaptogenesis, neuronal migration, and neocortical expansion in the human lineage. One of the regions with the highest proportion of duplicated sequence is the 22q11.2 locus, carrying eight LCRs (LCR22-A until LCR22-H), and rearrangements between them cause the 22q11.2 deletion syndrome. The LCR22-A block was recently reported to be hypervariable in the human population. It remains unknown whether this variability also exists in non-human primates, since research is strongly hampered by the presence of sequence gaps in the human and non-human primate reference genomes. To chart the LCR22 haplotypes and the associated inter- and intra-species variability, we *de novo* assembled the region in non-human primates by a combination of optical mapping techniques. A minimal and likely ancient haplotype is present in the chimpanzee, bonobo, and rhesus monkey without intra-species variation. In addition, the optical maps identified assembly errors and closed gaps in the orthologous chromosome 22 reference sequences. These findings indicate the LCR22 expansion to be unique to the human population, which might indicate involvement of the region in human evolution and adaptation. Those maps will enable LCR22-specific functional studies and investigate potential associations with the phenotypic variability in the 22q11.2 deletion syndrome.

## Introduction

Segmental duplications or low copy repeats (LCRs) constitute over 5% of the genome (Numanagiæ et al., 2018) and are complex patchworks of duplicated DNA fragments varying in length with over 90% sequence identity (Bailey et al., 2001, 2002a). This high sequence homology has so far impeded the accurate mapping and assembly of these regions in the human reference genome (Chaisson et al., 2015; Vollger et al., 2019). Although it has become evident that assembly using short read sequencing is unable to resolve these complex regions, some LCRs are often too long and complex even for more recently developed long read technologies to resolve (Genovese et al., 2013; Chaisson et al., 2015). In addition, large structural variation amongst haplotypes complicates the assembly of these LCR containing regions (Levy-Sakin et al., 2019). As a consequence, LCRs remain poorly mapped and characterized, despite their functional importance in evolution and disease.

The impact of these LCRs on primate and human evolution is increasingly recognized (Dennis and Eichler, 2016; Dennis et al., 2017). It is estimated that the origin of the LCRs coincide with the divergence of New and Old World Monkeys, 35–40 million years ago (Bailey and Eichler, 2006). However, a genomic duplication burst was observed in the great ape lineage, creating lineage-specific LCRs which are highly copy number variable (Marques-Bonet et al., 2009b). These LCR-containing regions in other great ape reference genomes are also enriched for gaps, since they are subject to similar assembly difficulties as those encountered in the assembly of these regions in the human reference genome (Mikkelsen et al., 2005; Gordon et al., 2016).

In humans, the 22q11.2 region contains a relatively higher proportion of LCRs compared with the rest of the genome. The origin of the human chromosome 22 LCRs (LCR22s) is concordant with the evolutionary timeline of LCRs in general. No duplicated orthologous LCR22 sequences are present in the mouse (Puech et al., 1997; Shaikh et al., 2000). The segmental duplication structure is present in the non-human primates, indicating the coincidence of their origin with the New-Old World Monkey divergence (Shaikh et al., 2000). FISH mapping of functional gene loci showed lineage-specific variation in the non-human primates, underlining the instability of the locus (Bailey et al., 2002b; Babcock et al., 2007). In addition, Guo et al. (2011) uncovered the presence of young Alu SINE elements at the boundaries of these expansions. However, data interpretation was performed against hg19, which harbors major differences in the LCR22 structure compared to hg38. In addition, techniques to resolve the exact structure of the LCR22s were lacking. Hence, *de novo* assembly of these haplotypes and interpretation against hg38 would provide a more accurate map of the evolutionary history of this complex locus.

Due to the high level of sequence identity, homologous segments within LCRs can misalign during meiosis, via a mechanism known as non-allelic homologous recombination (NAHR), resulting in genomic rearrangements including deletions, duplications, and inversions (Gu et al., 2008). The eight LCR22 blocks are referred to as LCR22-A until -H (McDonald-McGinn et al., 2015). NAHR between these LCR22s underlies the formation of the recurrent deletions associated with the 22q11.2 deletion syndrome (22q11.2DS) (MIM: 188400/192430), as well as the reciprocal duplications of this region often associated with abnormal phenotypes (MIM: 608363) (McDonald-McGinn et al., 2015). In 90% of the patients, the NAHR occurred between the largest LCR22-A and -D blocks, although other rearrangements exist (Burnside, 2015; Guo et al., 2018; Vervoort et al., 2019). The 22q11.2DS is the most common microdeletion disorder in humans and is characterized by congenital heart defects, immunodeficiency, hypoparathyroidism, and many other medical manifestations (McDonald-McGinn et al., 2015). However, the number and severity of symptoms is variable across patients.

We demonstrated hypervariability in the organization and the copy number of duplicons within LCR22s, especially LCR22-A (Demaerel et al., 2019). By combining fiber-FISH and Bionano optical mapping we assembled the LCR22s *de novo* and uncovered over 30 haplotypes of LCR22-A, with alleles ranging in size from 250 to 2,000 kb within 169 normal diploid individuals (Demaerel et al., 2019). Pastor et al. recently expanded the LCR22-A catalog by haplotyping the complete alleles of 30 22q11.2DS families (Pastor et al., 2020). To determine whether this extreme haplotype variability is human-specific, we set out to chart the inter- and intra-species variability of these LCR22s in non-human primates (Supplementary Table 1). The LCR22 structures of the great apes, including five chimpanzees (*Pan troglodytes*), one bonobo (*Pan paniscus*), two gorillas (*Gorilla gorilla* and *Gorilla berengei graueri*), six orangutans (*Pongo pygmaeus* and *Pongo abelii*), and one rhesus monkey (Old World Monkey, *Macaca malutta*) were analyzed by using a LCR22-specific fiber-FISH. To map the broader region, one representative of each species was analyzed by Bionano optical mapping. Interpreting these optical mapping data against the existing reference genomes enabled us to correct assembly errors and close gaps in the syntenic 22q11.2 loci. We demonstrate the non-human primate haplotypes to be less complex compared to humans. No intra-species variability similar to humans was observed suggesting that the hypervariability of the human LCR22-A haplotype is of recent origin.

## Materials and Methods

### Sample Collection and Cell Culture

Four chimpanzee samples (*Pan troglodytes* 7, 8, 15, and 17), one gorilla cell line (*Gorilla gorilla* 1), and five orangutans (*Pongo pygmaeus* 6, 7, 8, 9, and 10) were kindly provided by Professor MR (University of Bari, Italy) or purchased via the Biomedical Primate Research Centre (BPRC, Rijswijk, The Netherlands). All these samples were Epstein-Barr virus (EBV) transfected cell lines and cultured according to standard protocols. One chimpanzee fibroblast cell line was purchased from the Coriell Cell Repository (AG 06939A). One gorilla fibroblast cell line (Gorilla Kaisi) was originally obtained from the Antwerp Zoo (Antwerp, Belgium). The orangutan fibroblast cell line and the rhesus monkey kidney cell line were obtained from the European Collection of Authenticated Cell Cultures (ECACC) Repository. One EBV cell line was established from bonobo Banya from the Planckendael Zoo (Mechelen, Belgium). More information on the samples is provided in Supplementary Table 1. Blood was obtained during regular health checks of the animals.

### Fiber-FISH

The LCR22-specific fiber-FISH is a targeted optical mapping method developed to *de novo* assemble the LCR22 haplotypes at subunit level. In contrast to techniques using shorter DNA fragments, fiber-FISH was shown to be capable of spanning the LCR22s (Demaerel et al., 2019). To cover large parts of these alleles, long DNA fibers were extracted from the cultured cell lines using the FiberPrep^®^ DNA extraction kit (Genomic Vision) and the DNA was combed onto coverslips. The slides were then hybridized with the LCR22-specific customized probe set (Supplementary Table 2; Demaerel et al., 2019), supplemented with Bacterial artificial chromosome (BAC) probes targeting the unique regions between the LCR22s (Supplementary Table 2). Probes were designed in the LCR22s based on the reference sequence and named after its original position, due to the repetitive nature of these segmental duplications, the probes will hybridize in other LCR22s as well. Probes were subsequently labeled with digoxigenin-dUTP (Jena Bioscience), fluorescein-dUTP (Jena Bioscience), biotin-dUTP (Jena Bioscience), or combinations of these, using the BioPrime DNA Labeling System (Thermo Fisher Scientific). Indirect labeling with Alexa Fluor 647 IgG Fraction Monoclonal Mouse Anti-Digoxigenin (pseudocolored blue, Jackson Immunoresearch), Cy3 IgG Fraction Monoclonal Mouse Anti-Fluorescein (pseudocolored green, Jackson Immunoresearch), and BV480 Streptavidin (pseudocolored red, BD Biosciences) detected the primary labeled probes. The slides were scanned by an automated fluorescence microscope (Genomic Vision) at three excitation levels, corresponding to the three fluorophores. Images were automatically compiled by the system. The slides were visualized in FiberStudio (Genomic Vision) and manually inspected for regions of interest. Based on matching colors and distances between the probes, alleles were *de novo* assembled.

To differentiate between probes of the same color in the non-human primate haplotypes (for example B1, B3, and D7 are all yellow), we performed color-changing experiments. Probes of the same color were re-labeled to another color (for example the yellow probes B1 to cyan and D7 to red) and the experiment was performed again using the normal probes (red, blue, cyan, magenta, green) and the re-labeled yellow probes. In that way, changes in the assembled patterns indicate the correct probe composition. These analyses were repeated for each species for the uncertain probe compositions (Supplementary Figures 8–11).

### Bionano Optical Mapping

Using Bionano optical mapping, maps are generated providing structural variant information by comparison against a reference genome. The *de novo* assembly is based on the detection of fluorescently labeled long DNA fibers. Due to the lengths of these fibers, large deletions, duplications, inversions, and translocations can be visualized. In addition, the 22q11.2 locus and associated structural variants can be interpreted in different species.

To obtain the optical mapping data, high-molecular weight DNA from one chimpanzee (*Pan troglodytes* 15), one bonobo (Bonobo Banya), one gorilla (*Gorilla gorilla* 1), one orangutan (*Pongo pygmaeus* 8), and the rhesus monkey was extracted using the SP Blood & Cell Culture DNA Isolation kit (Bionano Genomics) and labeled using the DLS DNA labeling kit (DLE-1 labeling enzyme, Bionano Genomics). Samples were loaded onto Saphyr Chips G2.3 (Bionano Genomics), linearized, and visualized using the Saphyr Instrument (Bionano Genomics), according to the Saphyr System User Guide. All analyses were performed in Bionano Access (Bionano Genomics). General quality assessment via the Molecule Quality Report uncovered N50 values of 227, 215, 231, 252, and 177 kb for the chimpanzee, bonobo, gorilla, orangutan, and rhesus monkey, respectively. First, a *de novo* assembly was performed against the most recent human reference genome hg38. The effective coverage was 191X, 182X, 185X, 71X, and 11X, for the chimpanzee, bonobo, gorilla, orangutan, and rhesus monkey, respectively. An extra *de novo* assembly was performed against chromosome 22 of the corresponding non-human primate reference genome (chromosome 10 in case of the rhesus monkey). Effective coverages reached 355X, 318X, 297X, 340X, and 302X, for chimpanzee, bonobo, gorilla, orangutan, and rhesus monkey, respectively. Structural variants could be detected at the genome-wide level in the generated circos plot. The 22q11.2 region was visually inspected for structural rearrangements by zooming in to this region and comparing the compiled haplotypes with the hg38 reference or the non-human primate reference.

## Results

### Assembly of Chromosome 22 and the 22q11.2 Locus in Non-human Primates

**FIGURE 1:**
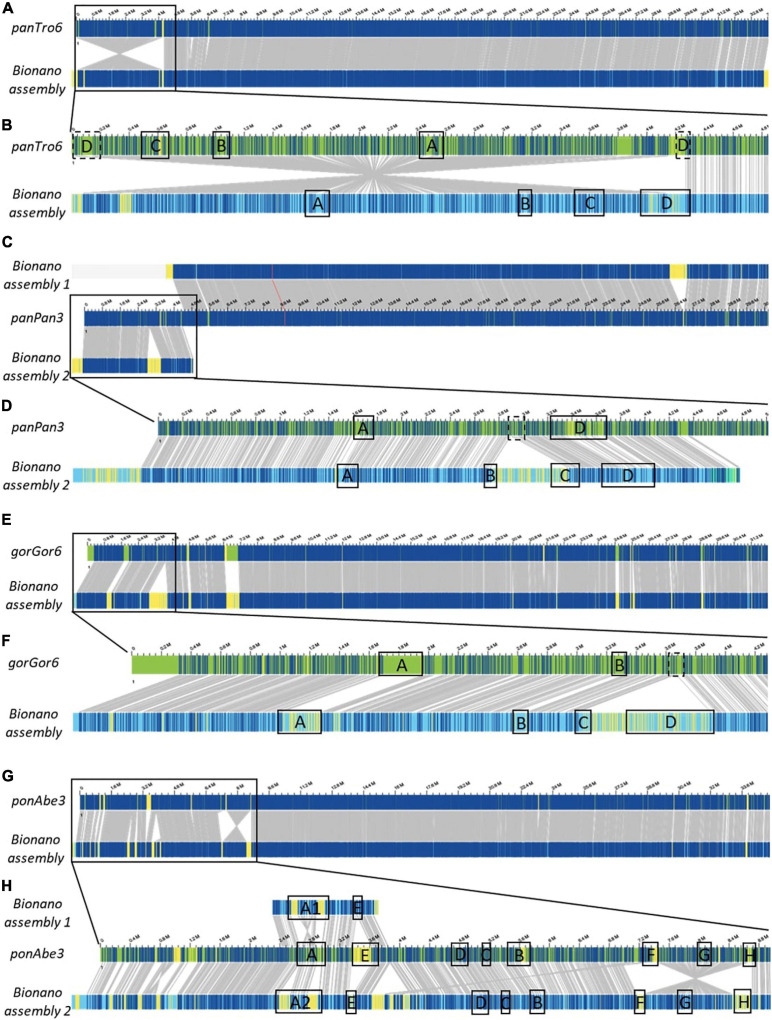
Bionano optical mapping comparison to chromosome 22 reference of Great apes. Chromosome 22-wide comparisons between the Bionano assembly and the reference of the chimpanzee (**A**, panTro6/Clint_PTRv2), bonobo (**C**, panPan3/Mhudiblu_PPA_v0), gorilla (**E**, gorGor6/Kamilah_GGO_v0), and orangutan (**G**, ponAbe3/Susie_PABv2). A zoom to the 22q11.2 locus is depicted for the chimpanzee (**B**, chr22:0-4,832,825), bonobo (**D**, chr22:0-5,007,102), gorilla (**F**, chr22:0-4,281,955), and orangutan (**H**, chr22:0-8,985,772), where the LCR22 blocks are represented. Blue labels in the Bionano optical maps represent aligned labels, and yellow labels unaligned. These unaligned labels are non-similarities between the investigated genomes. They can be present either at the nucleotide level or in very complex regions as segmental duplications. Gray lines between the maps indicate orthologous loci.

The great apes reference genomes contain several assembly gaps in chromosome 22 and especially the human 22q11.2 syntenic region. To assess the accuracy of the assemblies and close the gaps, we randomly selected one representative of the chimpanzee, bonobo, gorilla, orangutan, and rhesus monkey to be processed by Bionano optical mapping. By imaging long fluorescently labeled DNA molecules (>150 kb) followed by *de novo* assembly and local haplotyping the organization of larger chromosomal regions can be visualized (Chan et al., 2018). Subsequently, the assembled chromosome 22 alleles were compared to their genomic reference sequences (Figure 1). Chromosomal locations of LCR22-A until –D in human and the other investigated species are provided in Supplementary Table 3.

The comparison of the Chimpanzee reference (chromosome 22 of panTro6/Clint_PTRv2, January 2018) with the assembled Bionano haplotype uncovered one large misassembly, visible as an inversion variant in the Bionano plot, including LCR22-A until –D (Figures 1A,B and Supplementary Figure 1). The genes GAB4 and CCT8L2, located at the centromere in human hg38, are present at the distal end of this misassembly in the chimpanzee reference. In addition, genes RIMBP3C and LRRC74B are delineating the proximal start in the chimpanzee reference, while they are part of LCR22-D in humans. Fiber-FISH using BAC probes RP11-165F18 (proximal LCR22-D) and RP11-354K13 (distal LCR22-D) (Supplementary Table 2) validates the optical mapping assembly. No large yellow labeled regions, which indicate inconsistencies between the Bionano assembly and the reference, or differences in length are noticed for the LCR22s specifically (Figure 1B), except for LCR22-D, which is also disrupted by the misassembly.

Optical mapping assemblies based on the bonobo genomic reference of chromosome 22 resulted in two contigs (chromosome 22 of panPan3/Mhudiblu_PPA_v0, May 2020) (Figure 1C). During the analysis, complex multi-path regions splitted into two maps, since they are prone to misassembly. The distal part of the second contig (upper band) is identical to the reference except for one insertion. The first contig (lower band and zoom in Figure 1D) represents the 22q11 syntenic locus. In contrast to the bonobo reference genome where a 550 kb region between LCR22-B and LCR22-C is missing, the optical map shows that this region is present and the LCR22 organization is identical to the chimp and human organization (Supplementary Figure 2). This missing sequence in the bonobo reference genome is actually present as a separate contig (chrUn_NW_023259866v1), but not integrated into the reference genome assembly. The BAC probe CH17-131N14 (inter LCR22-B/C) in the fiber-FISH assay validated the Bionano assembly.

The optical map of Gorilla (Figure 1E, lower band) is largely corresponding with the genomic reference of chromosome 22 (upper band, chromosome 22 of gorGor6/Kamilah_GGO_v0, August 2019), except for the human 22q11.2 syntenic region (Figure 1F). In contrast to the reference, our optical map includes a 900-kb insertion, which contains the region between the LCR22-C and LCR22-D allele (Supplementary Figure 3). Part of the sequence of this region is represented in an unassembled contig chrUn_NW_022154665v1 of the gorilla reference genome. This contig contains the genes PI4KA, SERPIND1, SNAP29, CRKL, AIFM3, and LZTR1, located in the human genome between LCR22-C and -D, has a length of ∼300 kb and therefore is incomplete. Based on the large LCR22-D expansions identified by our fiber-FISH assemblies, it can be postulated that the remainder of the 900 kb insertion is LCR22-D sequence.

Although the distal part of chromosome 22 of the orangutan reference genome (ponAbe3/Susie_PABv2, January 2018) is generally consistent with the Bionano assembly (Figure 1G), some inconsistencies are visible in the proximal part (Figure 1H). The reference genome predicts the following LCR22 composition: A-E-D-C-B-F-G-H. However, in comparison to this reference, the orientation between LCR22-F and –H switched, visualized as an inversion variant between the reference and the assembly (Figure 1H). Individual Bionano reads validate the presence of the LCR22-F/H inversion (Supplementary Figure 4), with a coverage of 75 and 100X of the proximal and distal inversion breakpoint, respectively. The general coverage over the LCR22 blocks is between 70X and 110X. In addition, the Bionano assembly was able to differentiate between two LCR22-A alleles that were found for this orangutan (Pongo pygmaeus 8, Supplementary Figure 5 and Supplementary Table 4).

The rhesus monkey assemblies generated based on our Bionano data were largely consistent with the chromosome 10 genome reference of the rhesus monkey which contains the 22q11.2 syntenic locus (Mmul10/rheMac10, February 2019). In contrast to the great ape syntenic 22q11.2 assemblies, not only this region was correctly mapped but also the LCR22s (Supplementary Figure 6). Some rearrangements were observed in the centromeric locus, which is characterized by the presence of sequence gaps.

Considering that the 22q11.2 syntenic optical maps were validated by fiber-FISH, that the new assemblies are concordant across the great apes including human and that some unassembled contigs suggest the existence of those regions in the references genomes, we postulate the novel assemblies provide a more accurate representation of the great ape reference genomes.

### Conservation of the Syntenic 22q11.2 Locus Compared to the Human Reference

**FIGURE 2:**
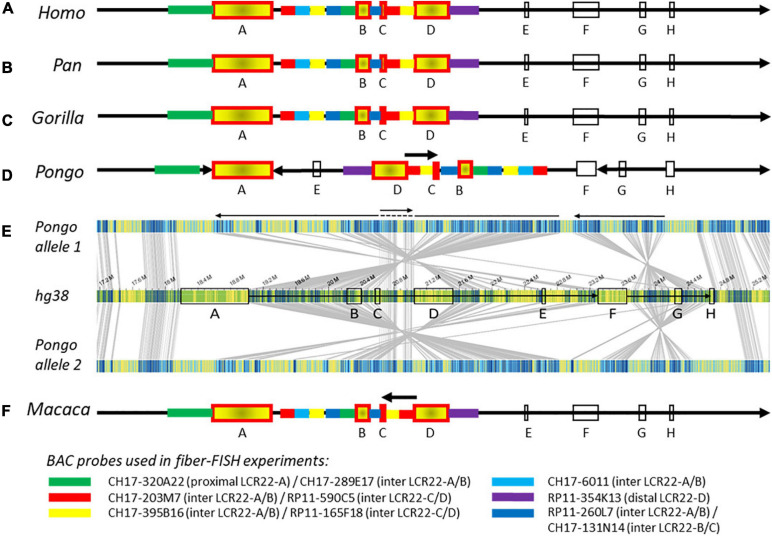
Composition of the 22q11.2 locus in human and non-human primates. Schematic representations of the 22q11.2 region, including LCR22-A through –H, based on Bionano optical mapping and fiber-FISH. As represented in **(A)** the human reference genome hg38, **(B)** chimpanzee and bonobo, **(C)** gorilla, and **(D)** orangutan. **(E)** Bionano optical mapping results of orangutan compared to the human reference genome. The middle bar represents the human hg38 reference genome with blocks indicating the LCR22s (corresponding to **A**). The top and bottom bar represent the assembled haplotypes for this orangutan. Gray lines between the maps indicate orthologous loci. Blue labels in the maps are aligned labels, and yellow labels unaligned. These unaligned labels are non-similarities between the genomes. They can be present either at the nucleotide level or in very complex regions as segmental duplications. Arrows below depict rearrangements between the human and the orangutan genomes. **(F)** Schematic 22q11.2 representation of the rhesus monkey, only based on fiber-FISH results. Chromosomal locations of the BAC probes can be found in Supplementary Table 2. Cartoons are not to scale.

To investigate the level of conservation relative to humans, the generated non-human primate optical maps were screened for rearrangements against the human reference genome. The resulting 22q11.2 syntenic assemblies were validated by fiber-FISH experiments using BAC probes targeting the regions flanking the proximal LCR22s (schematic representation in Figure 2A and Supplementary Table 2). Due to the low mapping rate between the rhesus monkey sample and the human reference genome, the Bionano analysis in this non-human primate could not be performed and the composition (Figure 2F) is only based on fiber-FISH results.

The order and organization of LCR22-A through -H in chimpanzee (Figure 2B and Supplementary Figure 7A), bonobo (Figure 2B and Supplementary Figure 7B), and gorilla (Figure 2C and Supplementary Figure 7C) is identical to human. In contrast, three large rearrangements were observed in the syntenic 22q11.2 locus of the orangutan (Figures 2D,E). First, the region between LCR22-F and -H, including LCR22-G, is inverted. Second, an inversion is present between the LCR22-A and -F blocks. Third, the orientation between LCR22-C and -D is not inverted compared with the human reference. This could be interpreted as an extra inversion between LCR22-C and -D following the rearrangement between LCR22-A and -F. The composition of these LCR22 blocks in the orangutan could also be derived from the Bionano assembly against the orangutan reference genome (Figures 1G,H). However, investigating this locus in the rhesus monkey by fiber-FISH uncovered the presence of this LCR22-C/D inversion, without the larger LCR22-A/F inversion (Figure 2F). Therefore, this LCR22-C/D inversion probably represents the ancient LCR22 block organization, while an inversion in a common ancestor of the other great apes created the haplotype as present nowadays in human. Hence, despite the unstable nature of the LCR22s themselves, the structural organization between the LCR22 blocks is conserved between gorilla, chimpanzee, bonobo, and human. Inversions, typically flanked by LCRs, are present in the orangutan and rhesus monkey haplotype.

### Evolutionary Analysis of LCR22-A

**FIGURE 3:**
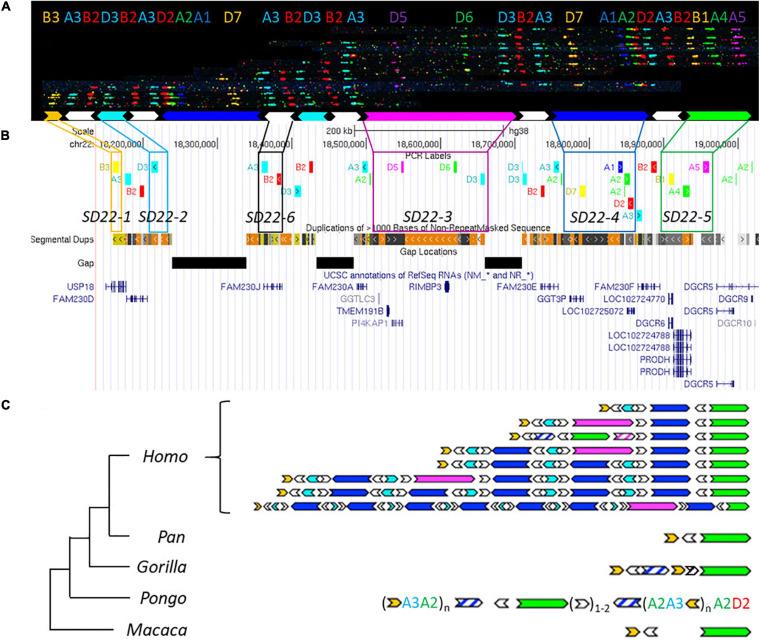
Human duplication structure and evolutionary analysis of LCR22-A. **(A)** *De novo* assembly of a LCR22-A haplotype. Individual fibers with probe patterns are collected during the analysis of the fiber-FISH slide and later compiled based on matching colors and distances between the probes. SD22 duplicons are assigned to specific probe combinations. **(B)** UCSC Genome Browser hg38 reference screenshot, with tracks for fiber-FISH probe BLAT positions, segmental duplications, gaps, and RefSeq genes. Assigned duplicons in **(A)** are decomposed to their corresponding fiber-FISH probes in this reference screenshot. **(C)** Evolutionary tree representation of the observed LCR22-A haplotypes. Only a subset of assembled haplotypes are depicted for human, to emphasize the human hypervariability. Filled, colored arrows represent copies of duplicons, and hatched arrows represent partial copies of duplicons of the same color.

The current reference genomes of great apes, except for the chimpanzee, are enriched for sequence gaps within the loci orthologous to the LCR22s. As a consequence, it was not possible to fully rely on the reference sequences and alleles had to be *de novo* assembled. For this, an LCR22-specific fiber-FISH method was applied, which has proven its value to resolve these complex structures in humans (Figure 3A; Demaerel et al., 2019). Exact probe identities were checked by changing the fluorophores of color-identical probes (Supplementary Figures 8–11).

Based on the extensive variability observed in the overall size and duplicon content of human LCR22-A (Figures 3B,C), we wanted to determine whether similar variation exists in the other great apes and rhesus monkey. Toward this end, five chimpanzees, one bonobo, two gorillas, six orangutans, and one rhesus monkey were analyzed (Supplementary Table 1). In contrast to the human variability, no structural variation was observed in any of the ten chromosomes of LCR22-A investigated in the chimpanzee samples (Supplementary Figure 12). In addition, both bonobo chromosomes had the same composition as those in the chimpanzee. This LCR22-A configuration (Figure 3C) is similar to the smallest haplotype observed in human suggesting that as the likely ancestor. However, this haplotype is rare in humans and only observed as a heterozygous allele in 5 of 169 human samples analyzed (Demaerel et al., 2019).

In the gorilla, the proximal and distal end are similar to the chimpanzee haplotype, except for a small insertion (Figure 3C). This is considered as a gorilla-specific insertion, since it is not present in the other non-human primate or human haplotypes. The same allele was observed in all four chromosomes of both gorilla cell lines. In addition to the large-scale rearrangements in the orangutan, we also observed major differences in the LCR22 compositions compared to the alleles of the other great apes (Figure 3C). First, the SD22-5 (green) duplicon, the distal delineating LCR22-A end in other great apes, is located in the middle of the allele, surrounded by SD22-6 duplicons. Second, tandem repeats of probe compositions (indicated between brackets in Figure 3C) characterize the proximal and distal end of the allele. This characteristic is different from the interspersed mosaic nature of the LCR22s in humans. In addition, structural variation is observed within these repeats in the six orangutan samples (Supplementary Table 3). Thus, the haplotypes observed in the orangutan are very different from those observed in other great apes (Figure 3C). In contrast, the rhesus monkey haplotype is mostly identical to the small chimpanzee haplotype composition, except for an ∼30 kb insertion of unknown origin separating the SD22-5 and SD22-6 duplicons.

In order to validate these results, we correlated the fiber-FISH data with the corresponding chimpanzee reference genome. The human locus chr22:18,044,268–19,017,737 including the LCR22-A allele, can be traced to the chimpanzee locus chr22:2,635,159–2,386,886 in the most recent reference genome (Clint_PTRv2/panTro6/January 2018). The fiber-FISH probe order predicted from this sequence exactly matches the obtained fiber-FISH pattern. Hence, this extra independent chimpanzee allele confirms the presence of a single LCR22-A haplotype in chimpanzee.

In conclusion, structural variation of the LCR22-A haplotype is observed in orangutans and humans, although different duplicons and structures are involved.

### Evolutionary Analysis of LCR22-B/C/D

**FIGURE 4:**
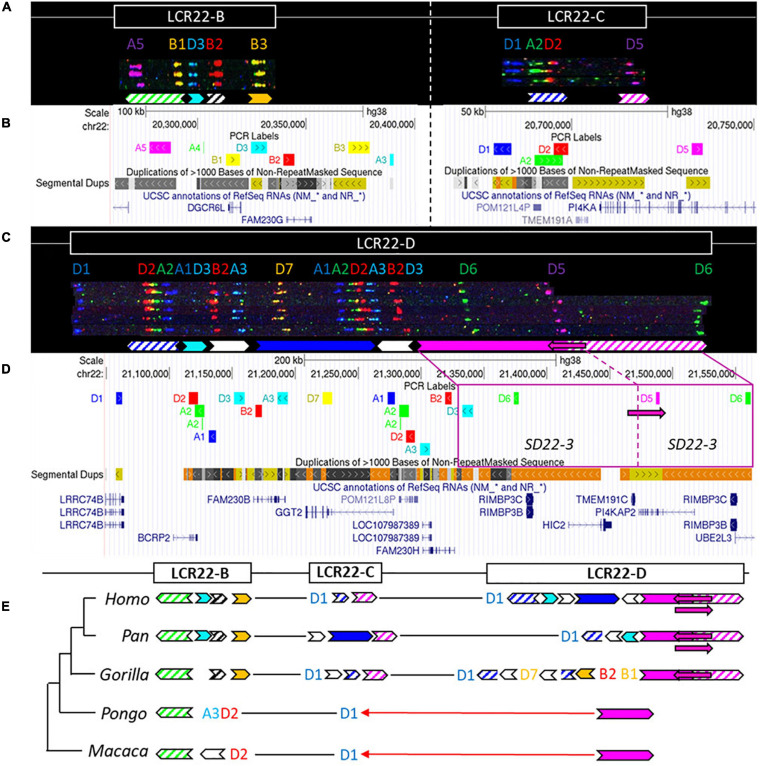
Human duplicon structure and evolutionary analysis of LCR22-B, -C, and –D. **(A)** *De novo* assembly of a LCR22-B (left) and LCR22-C (right) haplotype. SD22 duplicons are assigned to specific probe combinations, based on the probe composition in LCR22-A (Figure 3A). **(B)** UCSC Genome Browser hg38 reference screenshot of LCR22-B (left) and LCR22-C (right), with tracks for fiber-FISH probe BLAT positions, segmental duplications, and RefSeq genes. **(C)** *De novo* assembly of an LCR22-D haplotype based on matching colors and distances between the probes. SD22 duplicons are assigned to specific probe combinations. **(D)** UCSC Genome Browser hg38 reference screenshot, with tracks for fiber-FISH probe BLAT positions, segmental duplications, and RefSeq genes. The extended SD22-3 duplicon is decomposed to the corresponding fiber-FISH probes in the reference genome. **(E)** Evolutionary tree representation of the observed LCR22-B, -C, and -D haplotypes. Filled, colored arrows represent copies of duplicons, and hatched arrows represent partial copies of duplicons of the same color.

While LCR22-A is hypervariable in human genomes, LCR22-B and LCR22-C showed no variations, and only six different alleles were observed for LCR22-D (Figures 4A–D and Supplementary Figure 13; Demaerel et al., 2019). To evaluate the evolution of these LCR22s and assess intra-species variation in non-human primates, we investigated the syntenic LCR22-B, -C, and -D haplotypes in great apes and rhesus monkey by fiber-FISH as well. Since LCR22-B and -C could be small and hard to distinguish above fiber-FISH noise, the probe set was supplemented with BAC probes flanking these LCR22s (Supplementary Table 2).

For LCR22-B, the chimpanzee and bonobo were identical to the human haplotype, while the gorilla haplotype was similar, with the deletion of one duplicon (SD22-2) (Figure 4E). In the orangutan, the distal part is substituted by two probes (A3-D2). An extra insertion between these two probes creates the haplotype of the rhesus monkey. LCR22-C carries lineage-specific insertions and deletions in the *Pan* and *Gorilla* genus, while in the orangutan and rhesus monkey it is reduced to only one probe (D1) (Figure 4E). The human LCR22-D haplotype is subjected to structural variation, mainly in the SD22-3 duplicon (Demaerel et al., 2019). One variant, an internal inversion (indicated by the magenta arrow in Figures 4C,D), is present in 37% of the human haplotypes. The same variant was observed in a heterozygous state in two LCR22-D chimpanzee chromosomes (Figure 4E and Supplementary Figure 12), suggesting this variant precedes the split of the human lineage. The proximal start and distal end were conserved in Gorilla, with extra insertions compared to the human and *Pan* haplotype (Figure 4E). No structural variation was found at the distal end in these four investigated chromosomes. The LCR22-D haplotype in orangutan and rhesus monkey is composed of only two probes (Figure 4E). To conclude, LCR22-B, -C, and -D haplotypes start to evolve toward their human structures in a common ancestor of *Gorilla, Pan*, and *Homo*, based on the very short haplotypes found in orangutan and rhesus monkey.

## Discussion

FISH mapping studies of metaphase chromosomes from great apes using 22q11.2 BAC probes and analysis of sequencing data had demonstrated the LCR22 expansion to precede the divergence of old and New world monkeys, and suggested species specific LCR22 variation had occurred during primate speciation (Shaikh et al., 2000; Bailey et al., 2002b; Babcock et al., 2007; Guo et al., 2011). However, the FISH studies were mainly focusing on interrogation of the copy number of a limited number of genic segments and sequencing analysis was inevitably interpreted against human reference genome 37 (hg19), carrying important inconsistencies compared to the most recent reference genome hg38. By *de novo* assembling the LCR22s using LCR22-specific probes in the fiber-FISH assay we resolved the haplotype composition in five chimpanzees, one bonobo, two gorillas, six orangutans and a rhesus monkey. This approach provides a paradigm to map complex genomic regions and will leverage larger scale analyses of the LCR22s. The evolutionary analysis of the complex segmental duplications on chromosome 22 in different members of each species reveals a human-specific expansion of the LCR22-A haplotype, subject to structural variation in the human population.

Human-specific expansions of LCR22s possibly introduced additional substrates for LCR22-mediated rearrangements which could result in genomic disorders associated with the 22q11.2 locus. As demonstrated by Demaerel et al. (2019), the region of overlap between LCR22-A and LCR22-D is within a long stretch of homology encompassing SD22-4 flanked by SD22-6 on both sides, where recombination was shown to have taken place in case of an LCR22-A/D deletion. This locus is not present in any of the LCR22 blocks of the *Pan* genus. Pastor et al. (2020) narrowed this region to SD22-6, the duplicon encompassing the *FAM230* gene member. Guo et al. (2015) predicted the rearrangement breakpoint was located in the *BCR* (Breakpoint Cluster Region) locus, present in the distal part of SD22-4 (end of arrow). This locus was present twice in the *Pan* haplotype, once in LCR22-C and once in LCR22-D, but in opposite orientation preventing recombination leading to deletions and duplications. In the human lineage, the prevalence of both SD22-4 and SD22-6 increases in LCR22-A and LCR22-D. Hence, human-specific expansion of the region likely increases the susceptibility of chromosome 22q11.2 to rearrangements, similar to observations made in other diseases resulting from LCR-mediated rearrangement (Sudmant et al., 2013).

Chimpanzee and Rhesus LCR22-A haplotypes were the smallest amongst the analyzed apes and monomorphic in all screened specimens. While the analyses of several independent individuals and subspecies of Pan and Gorilla suggest that intraspecies variability is absent or limited, having access to a larger population of great ape specimen would strengthen such a conclusion. Because the endangered species act does not allow exchange of great ape tissue material between the United States and other countries, such analyses have been hampered. The small LCR22-A haplotype is likely the ancestral haplotype, with lineage-specific insertions and deletions. This ancestral haplotype is composed of three core duplicons (SD22-1, SD22-6, and SD22-5). Compared with most human haplotypes, three other core duplicons are missing (SD22-2, SD22-3, and SD22-4). These elements are present in, respectively, LCR22-B/D, LCR22-D, and LCR22-C of the *Pan* genus. Babcock et al. (2003) presented a model of insertion of duplicons into LCR22-A combining homologous recombination in the absence of a crossover with non-homologous repair. The model was proposed for an interchromosomal recombination, but can be applied for intrachromosomal events as well. Following insertion in the LCR22-A block, allelic homologous recombination is a possible mechanism for the creation and expansion of new haplotypes. Since *Alu* elements are frequently delineating LCR blocks in general and on chromosome 22 specifically, they form a perfect substrate for this type of rearrangements (Babcock et al., 2003; Bailey et al., 2003; Guo et al., 2011).

Structural variation of LCR22-A was observed in the orangutan lineage as well (Figure 3C). In the investigated samples, the main variation was present in the number of the repeat units at the proximal start and the distal end of the haplotype, consisting of the SD22-1 (human) duplicon and probes A3 and A2. However, the copy number of this yellow duplicon is fixed in the human population whereas other duplicons are subjected to copy number variation, e.g., SD22-3, SD22-4, SD22-2, and SD22-6. Therefore, other genes will be subjected to changes in the copy number and the expansion in combination with the structural variation observed in humans can be considered human-specific (Supplementary Table 5).

This study provides the hitherto highest resolution map of the LCR22s across our closest evolutionary relatives, improving the accuracy of the most recent non-human primate reference genomes (Figure 1). Although the reference assembly was in some species based on Bionano optical mapping data as well, use of different labeling enzymes (BspQ I vs. DLE-1) could explain inconsistencies between the assemblies. Bionano optical mapping identified three LCR22-mediated inversions in the orangutan lineage, and one in the rhesus monkey. A previous study focusing on the identification of inversion variants between human and primate genomes, observed the inversion between LCR22-C/D in the rhesus monkey, but was not able to identify any in the orangutan (Catacchio et al., 2018). The heterozygous inversion within the distal end of LCR22-D, present in humans and chimpanzee (Supplementary Figure 12), was previously identified by Bionano optical mapping in the chimpanzee, supporting the presence of this polymorphism in the chimpanzee population (Kronenberg et al., 2018). The extreme LCR22 amplification in gorilla, as described by Babcock et al. (2007), was not identified in this study. It seems likely that some of the LCR22 duplicons are amplified at other regions in the gorilla genome. Since metaphase and interphase FISH studies have a lower level of resolution, the exact location of these amplifications is not known but some amplifications appear to be located at telomeric bands. Hence, they will not be identified by our LCR22 targeted fiber-FISH analysis.

It remains to be uncovered whether this LCR22 variability influences the human phenotype, which elements are under selective pressure or rather the expansion is due to genetic drift. Human-specific expansions were also observed in LCRs present on other chromosomes that are known to cause genomic disorders (Boettger et al., 2012; Antonacci et al., 2014) and are possibly associated with human adaptation and evolution (Dennis and Eichler, 2016). For example, human-specific BOLA2 duplications on chromosome 16p11.2 and variation of DUF1220 domains on chromosome 1q21 are associated with iron homeostasis (Giannuzzi et al., 2019) and brain size alterations (Dumas et al., 2012), respectively. Gene duplications are a source for transcript innovation and expansion of the transcript diversity due to exon shuffling, novel splice variants, and fusion transcripts by the juxtaposition of duplicated subunits (Nuttle et al., 2016; Dougherty et al., 2017; McCartney et al., 2019). The human-specific *SRGAP2C* gene on chromosome 1 is an example of neofunctionalization (Dennis et al., 2012). The LCR-located gene, created by incomplete duplication, exerts an antagonistic effect on the ancestral *SRGAP2A* transcripts, resulting in human-specific neocortical changes (Charrier et al., 2012; Dennis et al., 2012). Another example is the partial intrachromosomal duplication of *ARHGAP11A* (chromosome 15) leading to *ARHGAP11B*, which is associated with brain adaptations during evolution (Florio et al., 2015). Hence, human-specific (incomplete) duplications of genic segments can render those genes into functional paralogs with possible innovating functions. These genes present evidence of positive selection and show a general increase in copy number in the human lineage (Marques-Bonet et al., 2009b).

The LCRs on chromosome 22 might be considered as an extreme source for expansion of the transcript catalog. Transcriptome studies may help to unravel the role of these human-specific expansions, since the presence of specific paralogs and their possible functional importance might be underestimated. Due to the duplicated nature of the LCR22s, paralogs share a high level of sequence identity. Therefore, short-read data are not always able to resolve the differences between transcripts arising from different paralogs and long read full-length transcriptome analysis will be required. In addition, tools to obtain the full-length sequences of the LCR22s and map the paralog variability will be essential to fully comprehend the extent of sequence variation present.

In summary, optical mapping of the LCRs on chromosome 22 unraveled lineage-specific differences between non-human primates and demonstrated the LCR22-A expansions and variability unique to the human population. It seems likely this expansion renders the region unstable and triggers NAHR resulting in the 22q11 deletions or duplications. To counter the paradox that LCR22 expansions reduce overall fitness, we hypothesize an important role for the region, previously described as the “core duplicon hypothesis” (Johnson et al., 2006; Jiang et al., 2007; Marques-Bonet et al., 2009a). Further research will be needed to unravel the functional importance of LCR22 expansion, including the role of paralog-specific transcripts.

## Data Availability Statement

Bionano raw and cmap files generated for this study can be found in the NCBI (National Center for Biotechnology Information) database (accession number PRJNA672266). Fiber-FISH data can be found in the Supplementary Figures.

## Ethics Statement

Ethical review and approval was not required for the animal study because blood to establish an EBV cell line was obtained during regular health checks of the animals.

## Author Contributions

LV and JV contributed to conception and design of the study and drafted the initial manuscript. LV conducted the experiments. LV and ND analyzed the data. ZP, OC, and MR provided cell lines to conduct the study. All authors contributed to manuscript revision, read, and approved the submitted version.

## Conflict of Interest

The authors declare that the research was conducted in the absence of any commercial or financial relationships that could be construed as a potential conflict of interest.

## Abbreviations

22q11.2DS: 22q11.2 deletion syndrome
BAC: bacterial artificial chromosome
LCR: low copy repeat
LCR22: low copy repeat on chromosome 22
NAHR: non-allelic homologous recombination.
