# Supplementary material for: 22q11.2 Low Copy Repeats Expanded in the Human Lineage
Source: Front Genet. 2021 Jul 15;12:706641. doi: 10.3389/fgene.2021.706641 (PMC8320366; doi:10.3389/fgene.2021.706641)
Supplement: Supplementary file 1 [file Data_Sheet_1.docx]

Supplementary Figures


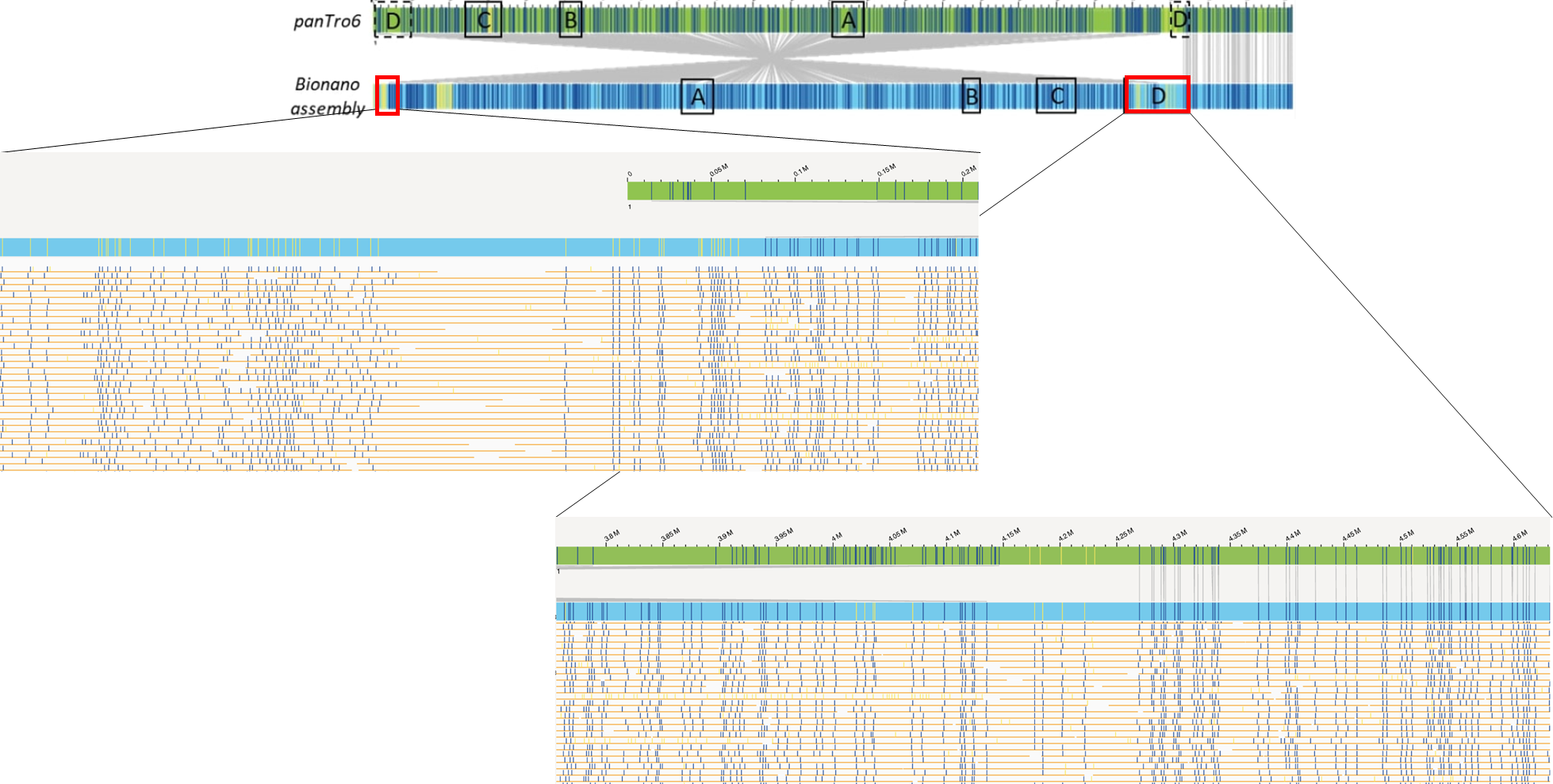


**Supplementary Figure 1.** Bionano molecule coverage of identified misassembly in the chimpanzee reference genome. Individual reads are shown for both ‘breakpoints’ of the scaffold to support the corrected Bionano assembly. Only part of the reads is visualized.


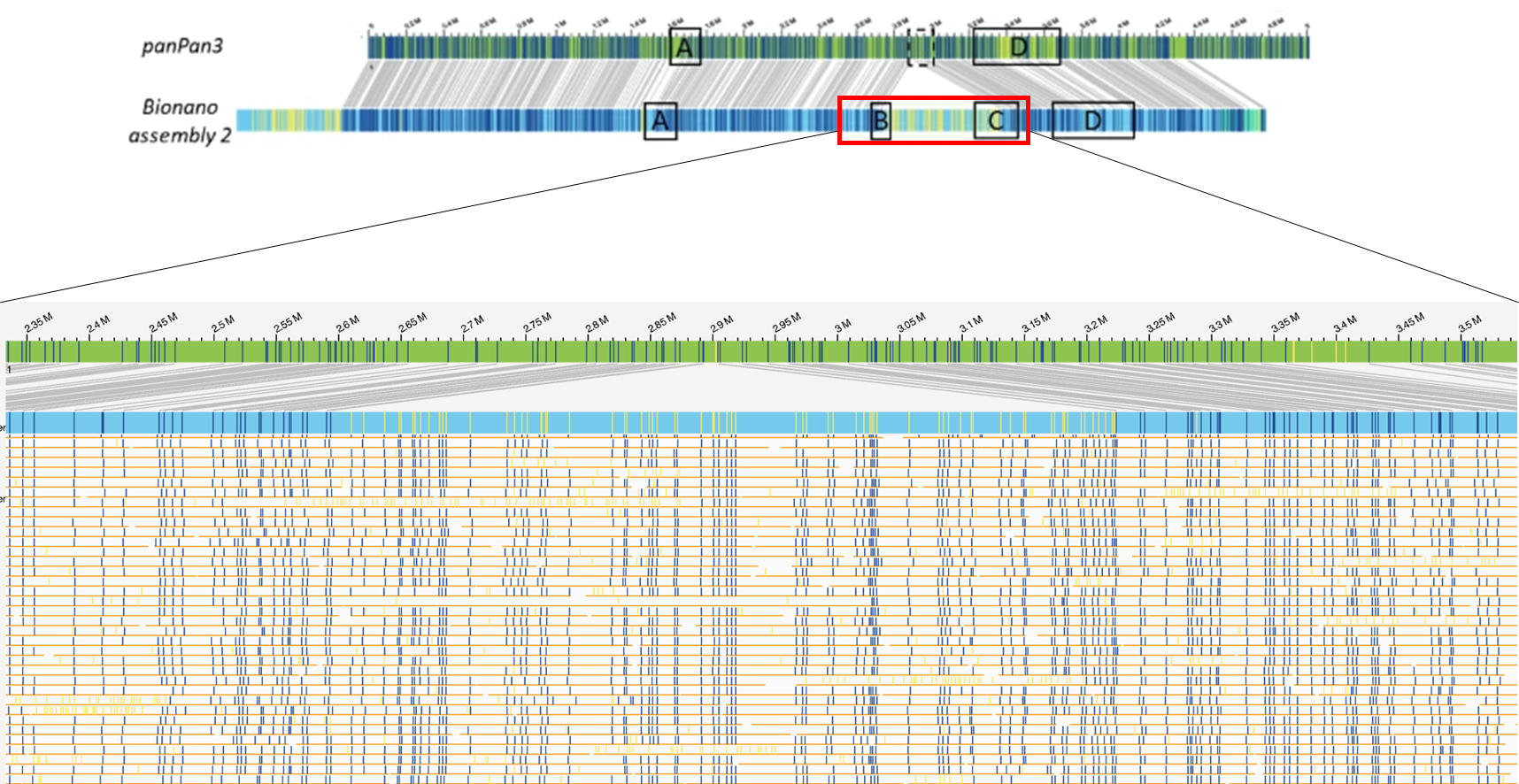


**Supplementary Figure 2.** Bionano molecule coverage of identified misassembly in the bonobo reference genome. Individual reads are shown over the ‘insertion’ to support the corrected Bionano assembly. Only part of the reads is visualized.


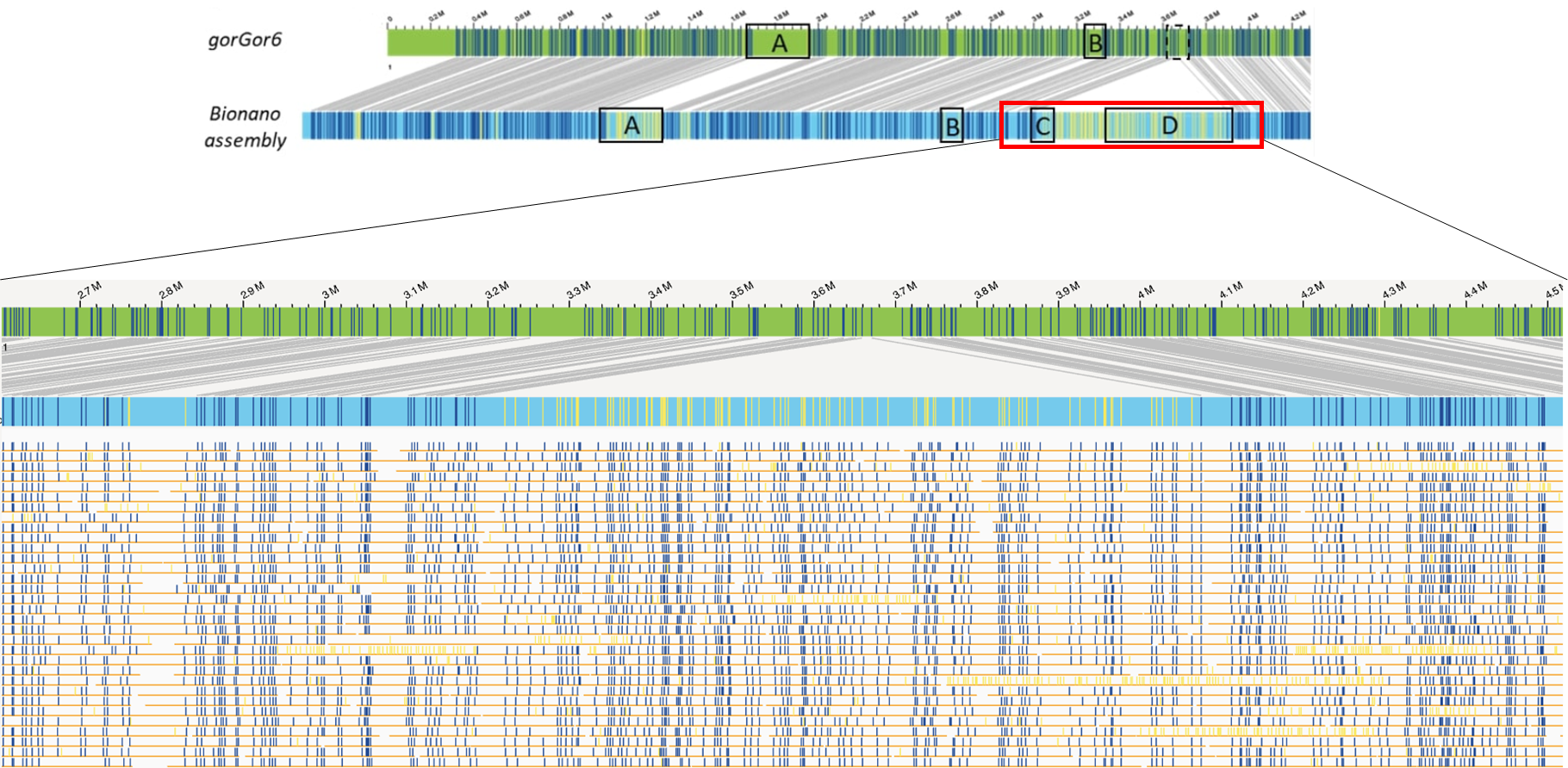


**Supplementary Figure 3.** Bionano molecule coverage of identified misassembly in the gorilla reference genome. Individual reads are shown over the ‘insertion’ to support the corrected Bionano assembly. Only part of the reads is visualized.


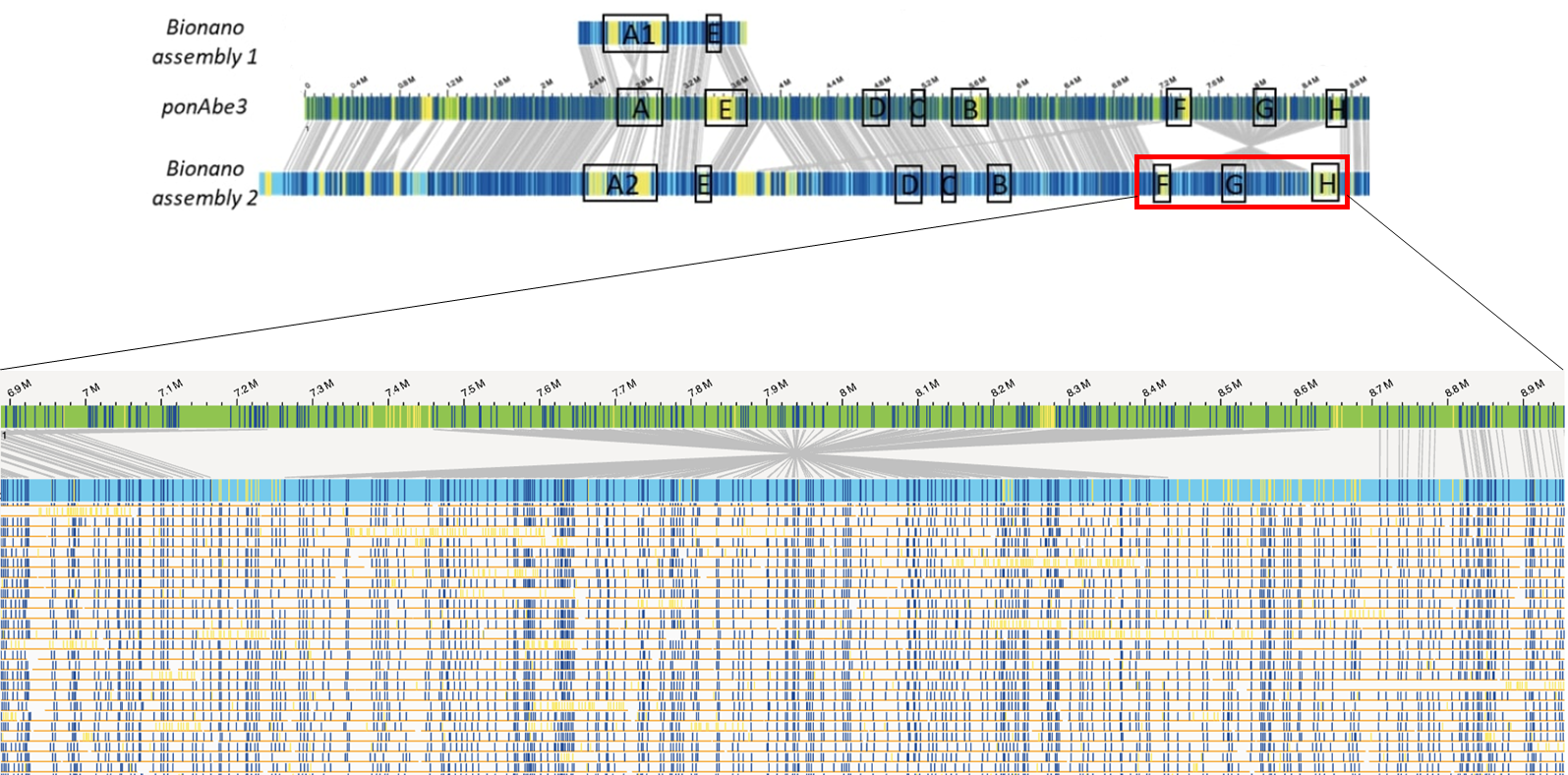


**Supplementary Figure 4.** Bionano molecule coverage of identified misassembly in the bonobo reference genome. Individual reads are shown over the ‘inversion’ to support the corrected Bionano assembly. Only part of the reads is visualized.


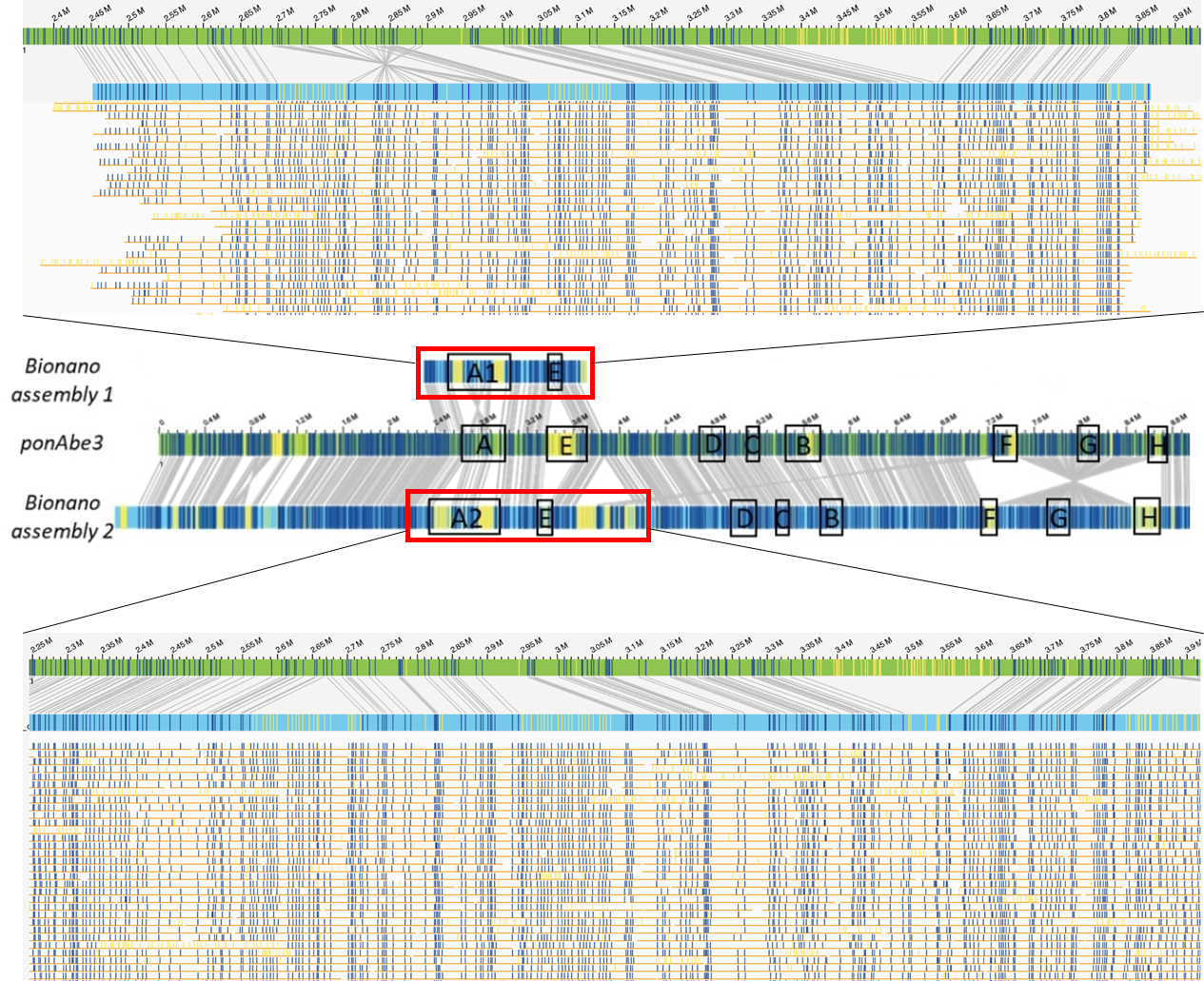


**Supplementary Figure 5.** Bionano molecule coverage of structural variation at the LCR22-A level in the investigated orangutan sample. Both haplotypes are supported by molecules spanning the locus.


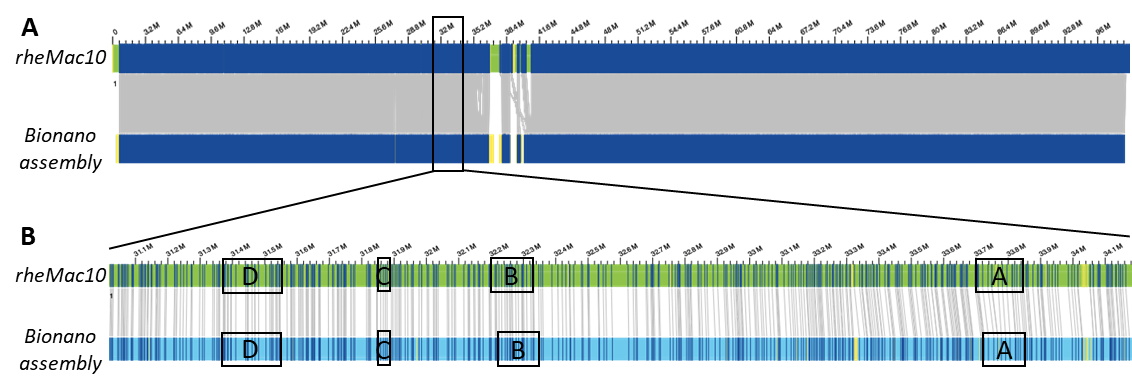


**Supplementary Figure 6.** Bionano optical mapping comparison to rhesus macaque reference genome (chromosome 10). (A) Chromosome 10-wide comparison between the reference genome chromosome 10 (Mmul_10/rheMac10, February 2019) and the assembled Bionano allele. Small rearrangements are visible around the centromere locus (36-40Mb). (B) Zoom to the syntenic LCR22-containing locus, without the presence of large rearrangements between the reference and the Bionano assembly.


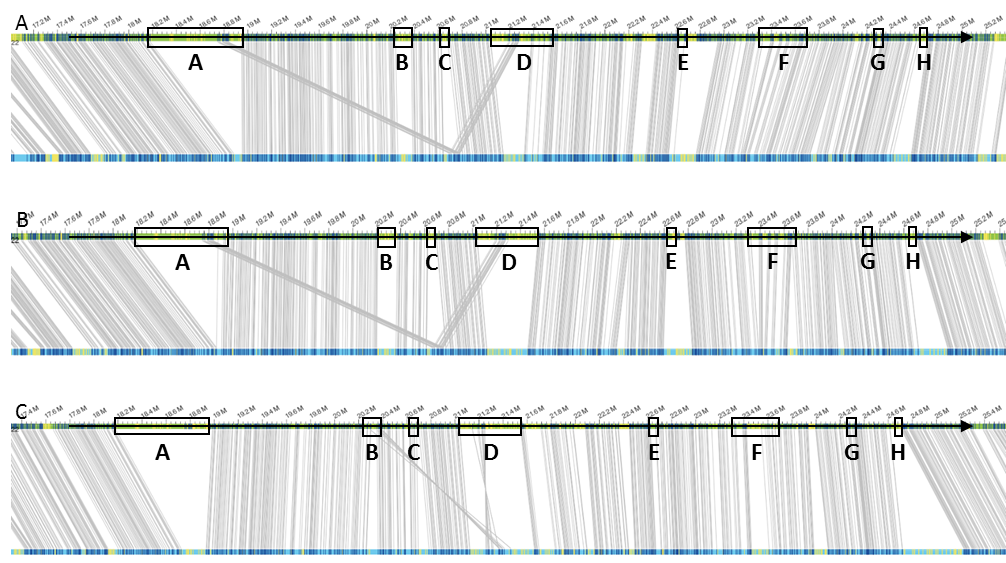


**Supplementary Figure 7.** Bionano optical mapping of the 22q11.2 region in chimpanzee, bonobo, and gorilla against the human reference genome (hg38). Regional organization of the 22q11.2 locus in (A) chimpanzee, (B) bonobo, and (C) gorilla. De novo assembled non-human primate maps are compared to the human reference genome (hg38). The top bar represents the human hg38 reference genome with blocks indicating the LCR22s. The bottom bar represents the assembled non-human primate haplotype. Grey lines between the maps indicate orthologous signals between them. Blue labels in the maps are aligned labels, and yellow labels unaligned.


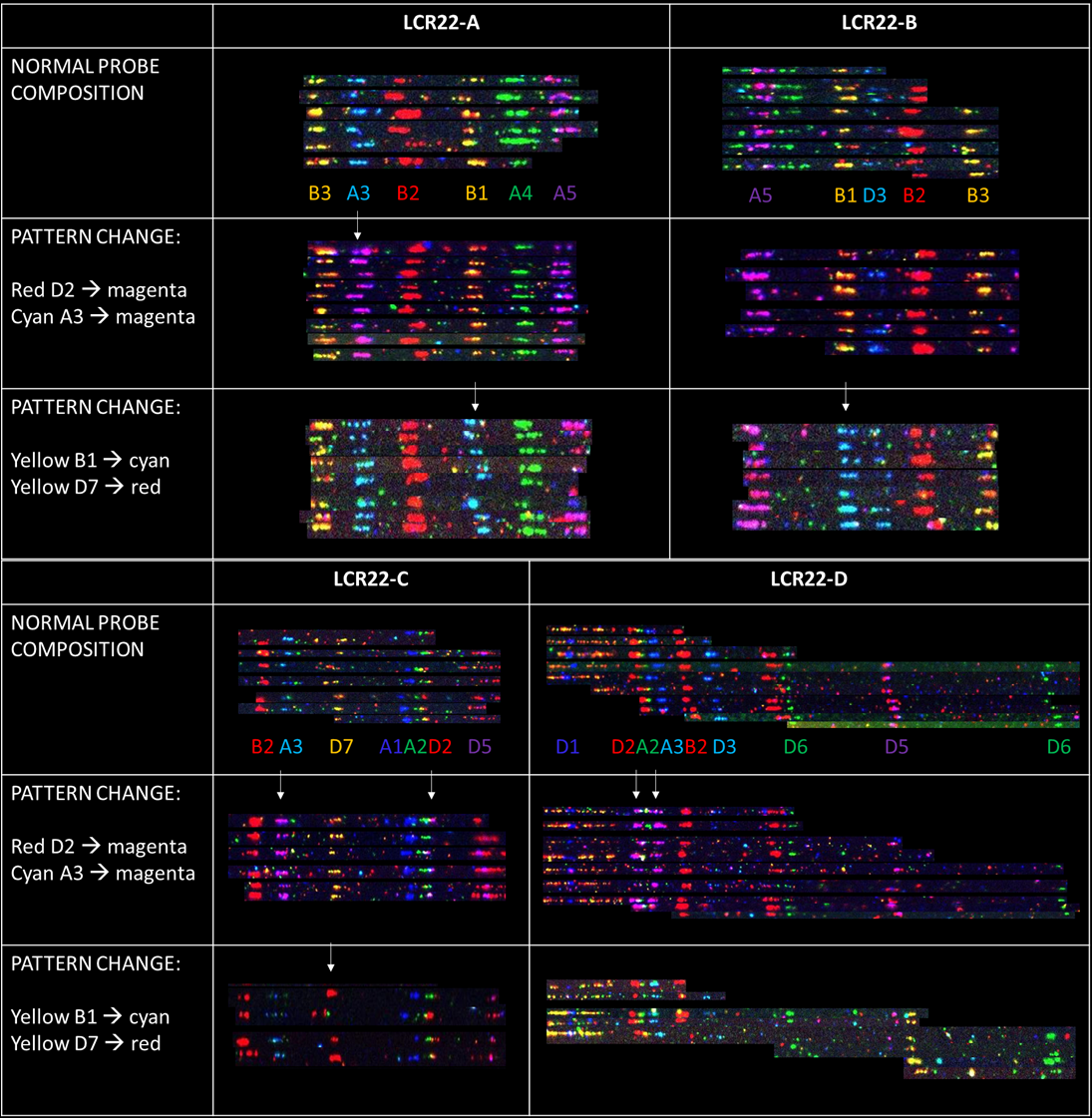


**Supplementary Figure 8.** Exact probe composition of the LCR22 chimpanzee and bonobo haplotypes. To derive the exact probe composition of the chimpanzee and bonobo haplotype, color-identical probes were differently labeled and hybridized to the slides. Changes of the pattern indicate the presence of the differently labeled probe. Red, cyan, and yellow probes were checked.


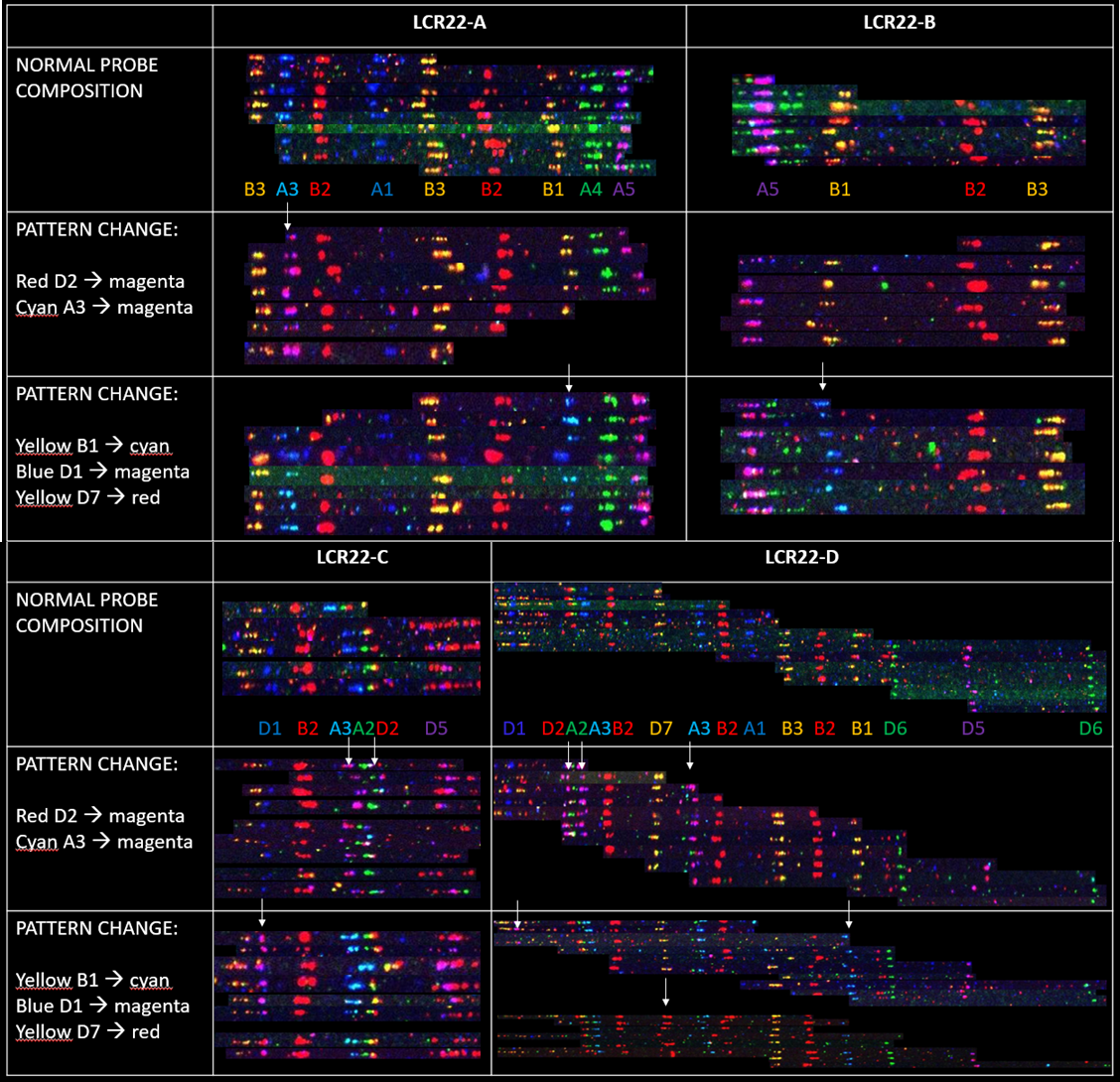


**Supplementary Figure 9.** Exact probe composition of the LCR22 gorilla haplotypes. To derive the exact probe composition of the gorilla haplotype, color-identical probes were differently labeled and hybridized to the slides. Changes of the pattern indicate the presence of the differently labeled probe. Red, cyan, blue, and yellow probes were checked.

*
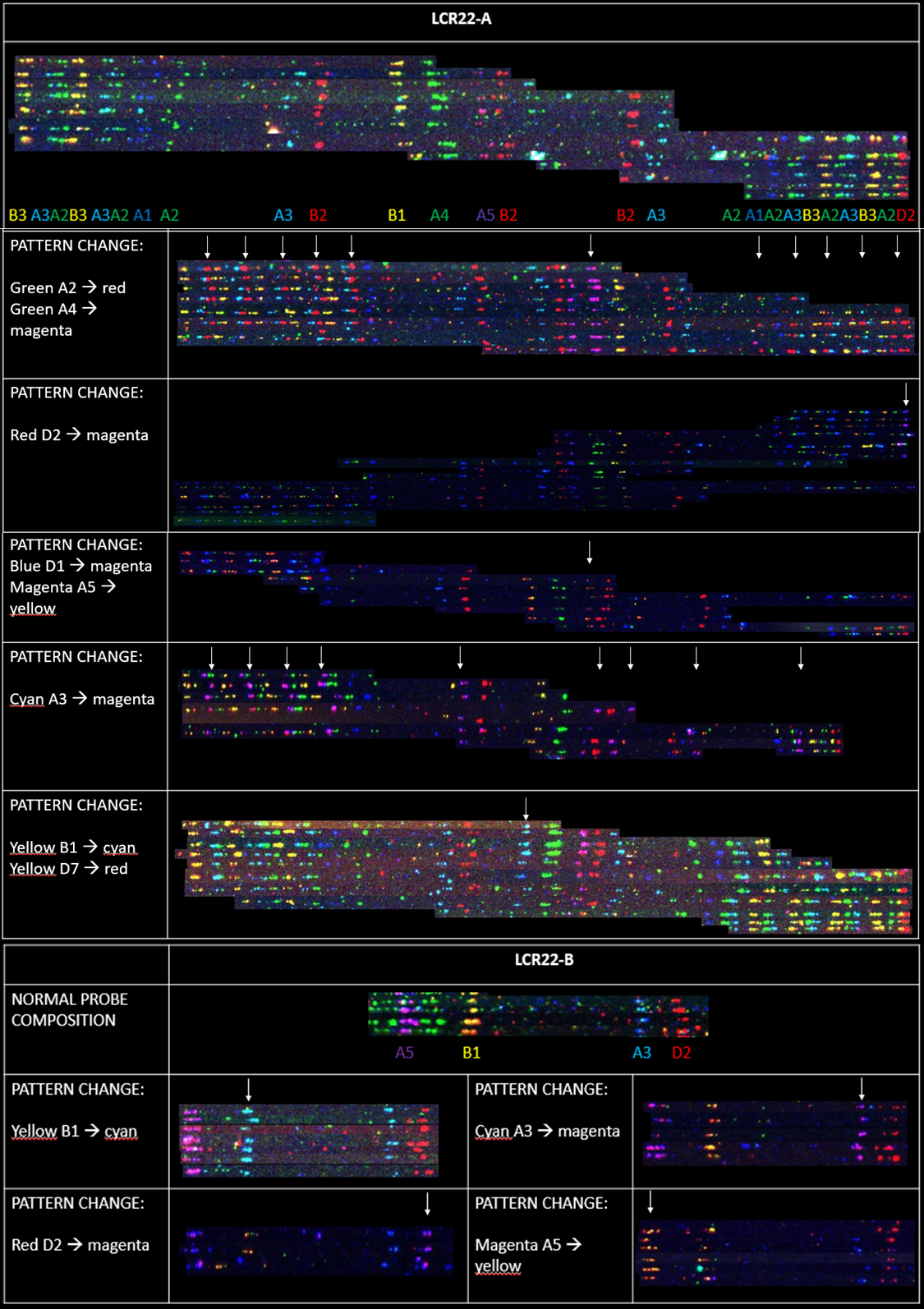
*

**Supplementary Figure 10.** Exact probe composition of the LCR22-A and -B orangutan haplotypes. To derive the exact probe composition of the orangutan haplotype, color-identical probes were differently labeled and hybridized to the slides. Changes of the pattern indicate the presence of the differently labeled probe. Red, cyan, blue, magenta, green, and yellow probes were checked. LCR22-C and -D are not included in the analysis, since they only consist of one and two probes, respectively. The probes are linked to unique BAC probes, predicting their composition.


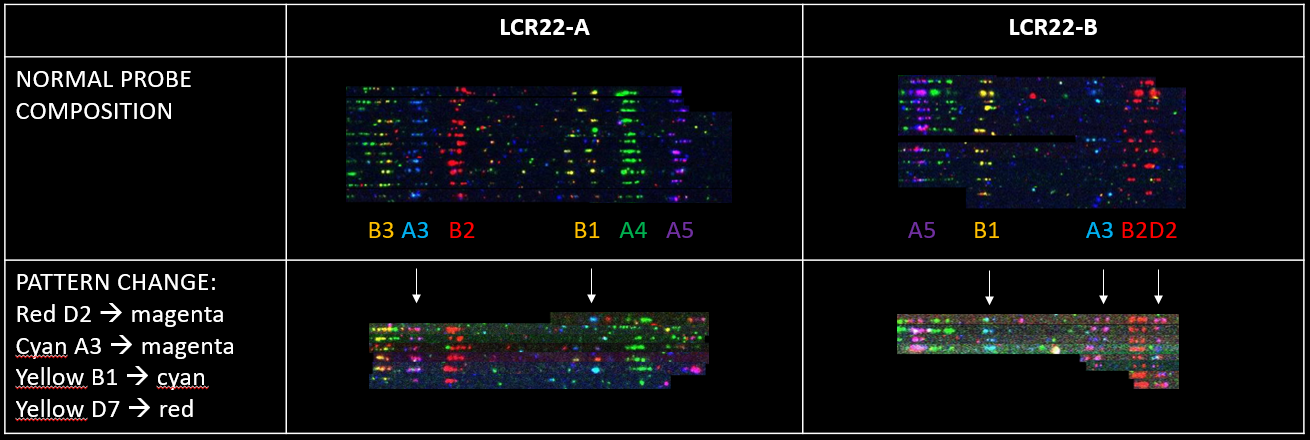


**Supplementary Figure 11.** Exact probe composition of the LCR22-A and -B rhesus macaque haplotypes. To derive the exact probe composition of the rhesus macaque haplotype, color-identical probes were differently labeled and hybridized to the slides. Changes of the pattern indicate the presence of the differently labeled probe. Red, cyan, and yellow probes were checked. LCR22-C and -D are not included in the analysis, since they only consist of one and two probes, respectively. The probes are linked to unique BAC probes, predicting their composition.


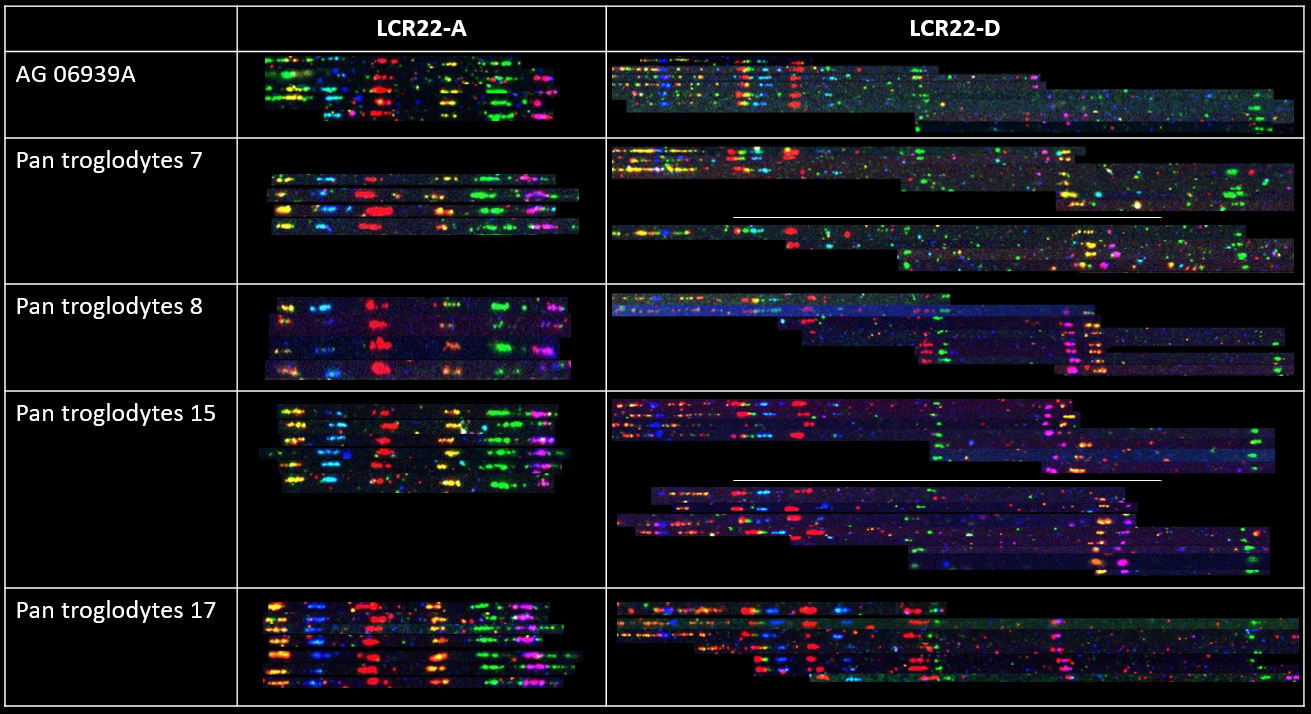


**Supplementary Figure 12.** Chimpanzee LCR22-A and -D haplotypes in investigated samples. *De novo* assembled haplotypes for LCR22-A and LCR22-D in the six investigated chimpanzees. Two chimpanzees (Pan troglodytes 7 and 15) showed structural variation distal in the LCR22-D haplotype. A white line distinguishes the two haplotypes. An extra probe (D extra, Supplementary Table 2) was added to the probe set of Pan troglodytes 7, 8, and 15 to distinguish between the


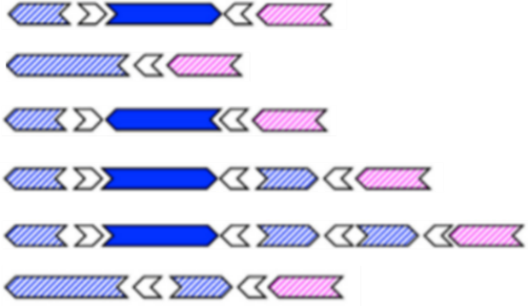


**Supplementary Figure 13**. Haplotypes of LCR22-D identified in the human population (adapted from Demaerel et al.)
